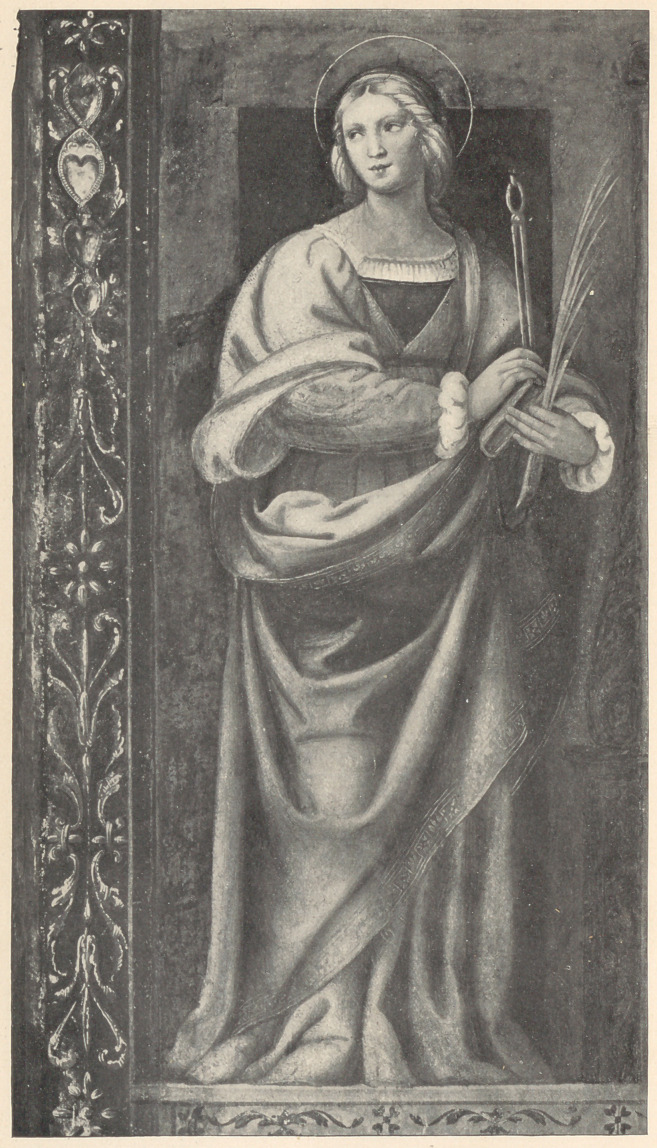# Apollonia, the “Patron Saint of Dentistry”

**Published:** 1900-01

**Authors:** C. N. Peirce

**Affiliations:** Philadelphia


### International Dental Journal.

Vol. XXI. January, 1900. No. 1.

### Original Communications.[1]

### APOLLONIA, THE "PATRON SAINT OF DENTISTRY."[2]

BY C. N. PEIRCE, D.D.S., PHILADELPHIA.

I have, by request, Mr. President, a very pleasurable function to perform. A lady member of this society, Dr. Mary H. Stilwell, desires to present to this organization the photograph of a character as unique and interesting as it is ancient, and she has kindly requested me to be her spokesman.

Some of you may be somewhat familiar with the subject to be presented, but doubtless to most of you the history and character of Saint Apollonia, the "Patron Saint of Dentistry," will be novel. That dentistry, of all the branches or specialties of medicine, has a patron saint I assume is known to very few in the dental profession. A brief recital of the history so far as obtainable of this saint has an historical as well as a professional interest. She was the daughter of a heathen magistrate in the city of Alexandria. Her mother, although not a Christian, was inclined to look with sympathy on the believers in that faith, and, being childless, she asked if the Virgin could grant her prayer for a child, on being told of her great power.

[1] The editor and publishers are not responsible for the views of authors of papers published in this department, nor for any claim to novelty, or otherwise, that may be made by them. No papers will be received for this department that have appeared in any other journal published in the country.

[2] Read before the Academy of Stomatology, Philadelphia, December 26, 1899.

She gave the pilgrims food and money, so, full of faith, she invoked Mary's intercession, after which the prayer was answered by the birth of Apollonia. To the child the mother often spoke of the wonderful power there was in the prayers of these people. It is not surprising, therefore, that Apollonia as she grew up felt more and more deeply that this alone was the one religion that could satisfy and ennoble her life. Longing to obtain the grace of baptism, she made her way to Saint Leonine, a disciple of Saint Anthony of Egypt, and as he baptized her he bade her go to Alexandria and preach the faith. So she went forth, and though she was only a woman, young and frail, yet so eloquent were her words, so fervent her zeal, that she made many converts. About this time a tumult had been stirred up in the city against the Christians, and the mass of the people were enraged at her preaching and came with bitter complaints to her father, who gave her up to be judged by the governor. They brought her before the idol temple and bade her worship the graven image. It is reported that she made a sign of the cross, and there came forth from the statue an evil spirit shrieking, "Apollonia has driven me hence." This was more than could be borne, the people thirsted for vengeance, so they tried by torture to overcome her constancy. She was bound, and one by one her teeth were drawn out, but still she did not flinch or fear, and on her refusal to accede to the demands of her persecutors and renounce her faith, she was brutally clubbed about the head and face and subsequently suffered death by fire.

For a period of nearly fifteen hundred years her intercession has been sought for relief from all pain incident to dental diseases, and her relics have been and are regarded as possessing great efficacy in the cure of the same.

The canonization of Saint Apollonia took place about the year 300 A.D.

On the 9th of February of each year she is commemorated. The so-called relics or remains of her head and jaws which were preserved from the fire into which she was thrown are preserved in various churches in the East and West. Church Saint Apollonia, at Rome, has a portion; in St. Maria Transtiberina her head rests; in St. Lawrence, outside the walls, her arms; in St. Basil's, part of her jaws; while in churches at Naples, Antwerp, Brussels, and Cologne, portions of the bones or teeth are cherished. In Quebec, Canada, we find also portions of a bone or tooth resting in some of the churches and viewed with veneration.

Furini has painted her in a picture now at Florence, Luini in

Monastery Maggiore Milan, and in the Milan Gallery there is an altar-piece by Francesco Granacce, on one wing of which is an almost life-size figure of her. Underneath the picture at Florence is the story of her life, from the moment of the angelic call until her death.

Procaccino has also painted her martyrdom, and she is also to be found in the works of that somewhat sentimental painter, Carlo Dolce.

Chapels and altars in her honor are found in many of the Eastern and Western churches. Her distinctive emblems are the pincers and tooth; the latter, in some of the paintings, is hung by a gold chain around her neck as an ornament.

Such records as we have been able to collect of Saint Apollonia give evidence of her dauntless courage, her perfect obedience to what she believed was the voice of God, and her fervent missionary spirit. Her story adds one more link to the long chain of heroes and heroines whose lives strike like sunlight across many a dark page of history.

The above short sketch has been obtained from "Gould's Lives of Saints," "Cassell's Dictionary of Religion," and a Letter of Dionysius to Fabius, Bishop of Antioch, published in the history of the Christian Church, by Eusebius, during the persecutions of the Christians at Alexandria, in the year 249.

## Figures and Tables

**Figure f1:**